# *Xylocopa sonorina* Smith, 1874 from Vancouver, British Columbia, Canada (Hymenoptera: Apidae, Xylocopinae) with comments on its taxonomy

**DOI:** 10.3897/BDJ.8.e49918

**Published:** 2020-04-14

**Authors:** Cory Sheffield, Jennifer Heron, Luciana Musetti

**Affiliations:** 1 Royal Saskatchewan Museum, Regina, Canada Royal Saskatchewan Museum Regina Canada; 2 British Columbia Ministry of Environment, Species Conservation Science Unit, Vancouver, Canada British Columbia Ministry of Environment, Species Conservation Science Unit Vancouver Canada; 3 The Ohio State University, Columbus, United States of America The Ohio State University Columbus United States of America

**Keywords:** large carpenter bees, non-native species, synonymy, DNA barcoding, distribution

## Abstract

**Background:**

Only one species of large carpenter bee, *Xylocopa virginica* (Linnaeus, 1771), has been recorded from Canada, albeit restricted to southern Ontario and Quebec. However, a single female specimen identified by Hurd in 1954 as *X. varipuncta* Patton, 1879 from British Columbia is in the C.A. Triplehorn Insect Collection at The Ohio State University (OSUC), suggesting that this species was accidentally introduced into coastal western Canada. As wood-nesters, many large carpenter bees are likely capable of expanding their range great distances by natural and unnatural transport methods while nesting inside suitable substrates, the presumed mode of transport into western Canada, and likely elsewhere. The ease at which the nests are transported has likely contributed to the nomenclatural and distributional ambiguity surrounding this species due to morphological similarities of specimens from North America, Hawaii, and several South Pacific islands.

**New information:**

By comparing DNA barcodes of specimens from the western United States to specimens from Hawaii, we confirm the early opinion of P.H. Timberlake (Timberlake 1922) that specimens long established on the Hawaiian Islands are the same *X. varipuncta* from continental North America. Furthermore, these DNA barcode sequences also match those of specimens identified as *X. sonorina* Smith, 1874 from the French Polynesian and Samoan Islands, thus fully supporting the opinion of Groom et al. (2017) that all are likely conspecific. As *X. sonorina*, a species described from and likely introduced to Hawaii is the oldest name available, *X. varipuncta* is here placed into synonymy. Additional research will be needed to trace the timing and pathway of introduction and establishment of *X. sonorina*; it is presumed that the species is native to the southwestern United States but has been established in Hawaii since the mid-1800s. It is also established in French Polynesia, the Samoan Islands, and likely other south Pacific islands, with additional records of occurrence from Java, New Zealand, and now Canada.

## Introduction

The large carpenter bees, genus *Xylocopa* Latreille, 1802 (Hymenoptera: Apidae, Xylocopinae), are large bumble bee sized bees that typically excavate nesting cavities into wood, bamboo, or the woody stems of plants (Hurd 1958a, Hurd 1958b, Hurd 1978a, Hurd 1978b, Gerling et al. 1989, Minckley 1998) though members of the subgenus Proxylocopa Hedicke, 1938 nest in the ground (Michener 2007). There are 32 species recorded from North America and Central America (Michener et al. 1994), many of these with recognized subspecies. Only one species has been previously recorded from Canada (Packer et al. 2007, Sheffield et al. 2017).

An interesting specimen of *Xylocopa* exists in the holdings of the C.A. Triplehorn Insect Collection at The Ohio State University (OSUC), a single female identified as *X. varipuncta* Patton, 1879, collected from Capilano Canyon near Vancouver, British Columbia by R.C. Osburn in 1949 (Fig. 1). The specimen was originally identified by P.H. Hurd Jr. in 1954 as *Xylocopa brasilianorum varipuncta* (Fig. 1d), the taxonomy likely following the classification of Ackerman (1916). Hurd (1955) later revised the genus *Xylocopa* occurring in California, but did not include the specimen from Canada in the distribution of *X. brasilianorum varipuncta* in that work. In later treatments (i.e., Hurd and Moure 1963) he considered *X. varipuncta* a valid species, separate from *X. brasilianorum* (Linnaeus, 1767). This unpublished Canadian record was also not included in the recent review of the subgenus Neoxylocopa Michener, 1954 (Mawdsley 2017) or in the checklist for the province of British Columbia (Sheffield and Heron 2019) due to its unlikelihood in Canada. This species is assumed to be native to the southwestern United States and Mexico (Hurd and Moure 1963, Mawdsley 2017).

Here we provide images of the specimen at OSUC, confirm that it was collected in British Columbia, and review how this species, though not established in Canada, could have made it to British Columbia and to other locations by natural and/or unnatural means. We also comment on the taxonomy of this species with consideration of recent studies and analysis using molecular methods, particularly DNA barcoding, that can assist traditional taxonomic and distributional work for an increasing number of bee species, and provide an up-to-date classification with a new synonymy.

## Materials and methods

To confirm that the specimen of *Xylocopa* was collected in British Columbia and not a mislabeled specimen, the OSUC collection was searched for other specimens collected by R.C. Osburn from Capilano Canyon at this approximate time (i.e. in 1949). It is assumed that finding multiple specimens from the same collecting event increases the likelihood that the material was not mislabeled.

Specimens were identified using the keys of Michener (2007) to subgenus, and Mawdsley (2017) for species. To supplement existing DNA barcodes for *Xylocopa* in the Barcode of Life Data (BOLD) System (Ratnasingham and Hebert 2007), including sequences from GenBank accessible through BOLD, we obtained sequences from recent material collected in the continental United States (Arizona), and material collected in Hawaii held in the collection of the Royal Saskatchewan Museum (RSKM). Procedures for obtaining DNA barcode sequences follow those provided elsewhere for North American bees (Sheffield et al. 2009, Sheffield et al. 2017). Sequences of *X. varipuncta* from North America and Hawaii generated here were compared to additional sequences from these areas and from other south Pacific Islands (Groom et al. 2017) using various sequence analysis tools on BOLD, including the Taxon ID Tree and Distance Summary tools.

## Taxon treatments

### Xylocopa (Neoxylocopa) sonorina

Smith, 1874

720FBA9B-6BE1-5A96-804E-1A2E9609DE79

Xylocopa sonorina Smith, 1874 in Smith 1874: 278 [♀] **Holotype** ♀. HAWAII, Sandw[ich]. Isl[and].” (not Sunda Isl.; see Lieftinck (1956)) [BMNH] [presented by E.W.H. Holdsworth Esq to the British Museum under register 1864.8 as per Lieftinck (1956)].
Xylocopa varipuncta Patton, 1879 in Patton 1879: 60 [♀] [**New Synonymy**] **Syntypes** ♀. USA, Arizona, by C.V. Riley [USNM]

#### Materials

- **Type status:** Other material. **Occurrence:** catalogNumber: OSUC 012135; sex: female; lifeStage: adult; previousIdentifications: *Xylocopa brasilianorum varipunctata* by Hurd '1954; **Taxon:** scientificName: *Xylocopa sonorina* Smith, 1874; kingdom: Animalia; phylum: Arthropoda; class: Insecta; order: Hymenoptera; family: Apidae; genus: Xylocopa; subgenus: Neoxylocopa; specificEpithet: sonorina; taxonRank: species; scientificNameAuthorship: Smith, 1874; **Location:** continent: North America; country: Canada; stateProvince: British Columbia; locality: Capilano Canyon; verbatimLocality: Capilano Can; **Identification:** identifiedBy: Cory S. Sheffield; dateIdentified: 2019; **Event:** eventDate: 18 June 1949; verbatimEventDate: vi-18-49; **Record Level:** type: PhysicalObject; institutionCode: OSUC; basisOfRecord: PreservedSpecimen

#### Distribution

Presumed native to the southwestern United States and adjacent Mexico (Mawdsley 2017), and introduced to the Hawaiian Islands, French Polynesia, Samoan Islands, Java, New Zealand and likely other south Pacific islands, the Marianas Islands, and now Canada.

## Analysis

We are confident that the specimen identified as *X. varipuncta* (Fig. 1) from British Columbia represents a valid record from Canada and is not mislabeled, as OSUC has several additional insect specimens collected by R.C. Osburn at Capilano Canyon from around that time (i.e., June to November 1949), including three Ichneumonidae, five specimens of *Tenthredo* (Symphyta, Tenthredinidae), and two specimens of *Trichiosoma triangulum* Kirby, 1837 (Symphyta, Cimbicidae). The latter species at least is widespread in North America, including ranging into British Columbia.

DNA barcodes from specimens of *X. varipuncta* generated in this study from western North America matched those from Hawaii, but also those from specimens in BOLD identified as *X. sonorina* Smith from the islands of Huahine-It and Mo’orea in French Polynesia, and Apia in the Samoan Islands, further supporting the opinion of Groom et al. (2017) that these are all conspecific, and confirming that this species was introduced to several Pacific Islands some time ago (i.e., Smith 1874, Cockerell 1919, Timberlake 1922). All specimens with full sequences have been assigned to Barcode Index Number (BIN; see Ratnasingham and Hebert (2013)) BOLD:ACE6828; mean genetic distance among specimens in this BIN is 0.52%; maximum genetic distance is 0.87%.

## Discussion

The taxonomy of *X. sonorina* has a long and interesting history, largely impacted by its arrival and subsequent establishment in Hawaii; in fact, the type locality of this species is Hawaii (Lieftinck 1956), well outside its suspected natural range. In North America, Ackerman (1916) considered *X. varipuncta* a synonymy of *X. brasilianorum* based on morphology of females only as the male of Patton’s (Patton 1879) species was unknown at that time, but considered it a distinct subspecies occurring in Arizona and southern California. Linnaeus’s (Linnaeus 1767) original, albeit brief description of *X. brasilianorum* indicated the type locality “Habitat in America”, but this vague geography was interpreted by Moure (2012) as probably meaning Rio de Janeiro, Brazil. Ackerman (1916) also recognized the widely distributed subspecies *brasilianorum* from North America (ranging from Texas, Arizona, southern California, and Mexico), south into Central and South America, and the West Indies, and also two additional subspecies, *aeneipennis* (De Geer, 1773) in Arizona, and *cubaecola* Lucas, 1857 in California. The latter two subspecies of Ackerman (1916) represent misidentifications as *X. aeneipennis* is now considered a valid species from South America (Suriname), and *X. cubaecola* is a valid species endemic to Cuba (Ospina 2000, Moure 2012). Hurd (1955) presumably treated these as valid species as neither was included in his key to subspecies of *brasilianorum* in America north of Mexico. Furthermore, Mawdsley (2017) also did not include *X. cubaecola* or *X. brasilianorum* as species of subgenus Neoxylocopa occurring in North or Central America. Timberlake (1922) felt that specimens of *Xylocopa* from the Hawaiian Islands (Honolulu) previously identified as *X. aeneipennis* by Smith (1879) and Blackburn and Kirby (1880) were the same as *X. varipuncta* from the continental United States, an opinion shared by T.D.A. Cockerell (as per Timberlake 1922), and later Williams (1927) and Nishida (1958); Blackburn and Kirby (1880) indicated that *X. aeneipennis* was common enough in Honolulu and elsewhere on the Hawaiian islands that it caused considerable damage to trees and timber. T.D.A. Cockerell (cited in Timberlake 1922) also felt that *X. varipuncta* was a distinct species from *X. brasilianorum*.

Though Hurd (1955) identified the specimen from British Columbia in 1954, he did not mention it or indicate a range for subspecies *varipuncta* extending further north than northern California; this specimen from Canada was also not mentioned by Mawdsley (2017). Hurd (1955) also indicated that material apparently identical to subspecies *varipuncta* had become established in Hawaii, presumably based on Timberlake (1922). Later still, Hurd and Moure (1963) recognized *X. varipuncta* as a valid species distinct from *X. brasilianorum* based on male genitalia. Hurd felt it was incorrect to call the material from Hawaii *X. varipuncta*, instead referring to it as *X. brasilianorum sonorina* Smith (Hurd 1958b; likely following Lieftinck (1956)) and *X. sonorina* (Hurd 1978a), a species originally described from the Sunda Islands, Indonesia (Smith 1874), though Lieftinck (1956) re-examined the type material and indicated that the type locality should be the Hawaiian Islands, noting a misreading of Sandw[ich]. Isl[and]. Lieftinck (1956) placed *X. varipuncta* (as *X. brasilianorum varipuncta*) into synonymy with *X. b. sonorina*.

The misinterpretation of the type locality for *X. sonorina*, and subsequent misidentifications of material from Hawaii has had serious implications. Though there is only one species of *Xylocopa* known from Hawaii (see Snelling 2003), Smith (1879) subsequently identified specimens from Honolulu (presumably the same species as his *X. sonorina* named five years previous) as *X. aeneipennis*, the species known from Suriname mentioned above. This error is likely why Timberlake (1922) also used the name *X. aeneipennis* for Hawaiian material (after Smith 1879, not De Geer 1773) and not *X. sonorina*, justifying his synonymy with the North America species *X. varipuncta*. Since Lieftinck (1956), additional authors have subsequently and correctly applied *X. sonorina* to specimens from Hawaii (e.g., Hurd 1958b, Hurd 1978a, Barrows 1980, Gerling 1982), though some have not (e.g., Tadauchi 1994). Hurd (1958b) called *X. brasilianorum sonorina* a Hawaiian carpenter bee, seeming to suggest that it was native to Hawaii, or at least non-native to the continental United States, but indicating that it had become adventive in the Marianas Islands (Krombein 1950, Tadauchi 1994) and that specimens had been also intercepted in shipments of wood in San Francisco (Hurd 1955) and Japan (Maidl 1912). Soon after, Hurd and Moure (1963) and others (e.g., Barrows 1980) did consider *X. sororina* a non-native species in the Hawaiian Islands, supporting Timberlake’s (Timberlake 1922) suggestion that species in Hawaii was possibly introduced to these islands; this was also considered likely by Snelling (2003). Timberlake (1922), Williams (1927), Nishida (1958), and Tadauchi (1994) all felt the Hawaiian species was the same as *X. varipuncta* from the continental United States; only Linsley (1966) applied the name *X. sonorina* as an American carpenter bee.

Michener (2007) indicated that *Neoxylocopa*, to which this species belongs, was the only subgenus of *Xylocopa* occurring in both Eastern and Western Hemispheres, albeit native to the Western Hemisphere and introduced through commerce to certain Pacific Islands. It is unfortunate that Leys et al. (Leys et al. 2000, Leys et al. 2002) did not have material identified as *X. sonorina* for comparison to *X. varipuncta* in their molecular phylogenetic studies, as the recent review of this subgenus in North and Central America considered *X. varipuncta* a valid species (Mawdsley 2017), but indicated that if the species on the Hawaiian Islands turns out to be conspecific with the North American form through the use of DNA barcoding or other molecular tools, *X. sonorina* would be the correct name. Groom et al. (2017) provided such support, comparing specimens of *X. sonorina* from Samoa originally identified as *X. varipuncta* (Groom et al. 2014), French Polynesia, Hawaii, and the continental United States, though only suggested that the names may be synonymous. Additional genetic data provided here adds further support for the conspecificity of specimens from in Hawaii and North America (*X. varipuncta*) which share DNA barcodes with specimens identified as *X. sonorina* from the islands of Huahine-It and Mo’orea in French Polynesia, and Apia in the Samoan Islands (Groom et al. 2017); *X. sonorina* is the oldest name available for this species, as suggested by Snelling (2003), Mawdsley (2017), and Groom et al. (2017). As such, we synonymize *X. varipuncta* under *X. sonorina*.

Following Lieftinck (1956), the Hawaiian type locality for *X. sonorina* is from an area where it was most likely introduced, but the species is likely endemic to the continental United States. Its occurrence in Hawaii and other south Pacific Islands are likely due to introductions that resulted in establishment, though other *Neoxylocopa* (native to the Western Hemisphere) are presumed native to the Galápagos (Cockerell 1935, Linsley 1966, Vargas et al. 2015) and Revillagigedo Islands (Hurd 1958a, Michener 2007). *Xylocopa darwini* Cockerell, 1926 is considered the only native bee species in the Galápagos (Hurd 1958a, Rasmussen et al. 2012, Vargas et al. 2015) and possibly first arrived in driftwood (Cockerell 1935). Hurd (1958a) suspected that *X. clarionensis* Hurd, 1958 of the Revillagigedo Islands was likely a recently derived form of *X. varipuncta* from the mainland, though did not speculate on how it crossed the 650 km to Baja California or 965 km to other areas of Mexico. Janzen (1964) found nests of *X. varipuncta* in a driftwood log on a beach in Mexico possibly supporting Cockerell’s (Cockerell 1935) explanation for *X. darwini*; though Janzen (1964) indicated that these were likely made after the log washed ashore, it is likely for driftwood to be carried back into the ocean and transported elsewhere.

It is entirely possible that a nest with at least one specimen of *X. sonorina* arrived in coastal British Columbia within lumber via commercial means, or less likely due to the distance, within driftwood (e.g. Cockerell 1935, Linsley 1966, Vargas et al. 2015) from the United States, and subsequently emerged and was captured. Short travel times of driftwood logs in ocean currents may promote short distance dispersal in wood-nesting bees, but would likely not support the long distance travel and survival from North America to the Hawaiian Islands, French Polynesia, and the Samoan Islands. However, the hypothesized < 1,000 km explanations for the natural arrival of *X. darwini* to the Galápagos, and *X. clarionensis* to the Revillagigedo Islands could be possible (Hurd 1958a). In ocean currents, some driftwood is capable of travelling great distances (e.g., Eggertsson 1993) provided it does not lose buoyancy, which typically can occur with 6 months to 1.5 years (Häggblom 1982), with logs of lesser volume (Eggertsson 1993) or greater density (Ruiz-Villanueva et al. 2014) with decreased buoyancy. For many animals, total travel time is likely the main issue for successful natural dispersal in oceans over great distances (see de Queiroz 2014). However, no species of *Xylocopa* is yet known to have multiyear diapause that would facilitate extensive long distance/time travel in wood; all such species winter as prepupae while members of the subfamily Xylocopinae typically overwinter in adult groups containing both males and females (reviewed by Danforth et al. 2019). Michener (Michener 1979, Michener 2007) indicated that few bee species are likely capable of natural long distance dispersal by flight, typically favouring larger species (e.g., Araújo et al. 2004, Greenleaf et al. 2007), partially explaining why most oceanic islands are naturally depauperate of apoid species. Longer distance dispersal by bees is more likely facilitated by nest transport, thus favouring species like carpenter bees that nest in wood or other movable substrates (Michener 1979), and smaller bees, such as *Hylaeus* Fabricius, 1793 (Colletidae) are probably more suitable for long distance travel than larger bees (*Michener 1979, Daly and Magnacca 2003*).

Much faster travel time of materials containing bee nests is obtained via commerce, which is likely the main means of introduction today. Recently, a carpenter native to Japan and China was found in California (Dahlberg et al. 2013), suggesting that commerce is likely important. There are also historic records of other native North American species of *Xylocopa* being intercepted at other locations; Hurd (1961) (citing Dover 1924) indicated that *X. virginica* was collected in Nottingham England from nests built in wood. Groom et al. (2017) suggest that early Polynesian seafarers could have transported wood containing nests to several islands at a rate that would promote establishment, but also suggested that more recent and purposeful introduction of this species for passionfruit pollination may have occurred. However, this species was introduced on several Pacific Islands some time ago (i.e., Smith 1874, Cockerell 1919, Timberlake 1922), though this species was not recorded in any of the South Pacific regions covered by Michener (1965). Incidentally, nests of smaller bee species would also be harder to detect in materials inspecition at international borders; in Canada, at least 20 species of wood- or stem-nesting bees, most of them small, have been introduced and established (see Sheffield et al. 2011, Gibbs and Dathe 2017, Martins et al. 2017, Normandin et al. 2017). Other larger species (i.e., *Megachile xylocopoides* Smith, 1853 (Megachilidae)) have been intercepted at the Canadian border, but have not been recorded as established (Sheffield et al. 2011).

Once arriving to a new location, some bee species can succeed even with very low numbers of colonizers. Zayed et al. (2007) showed that some bee species may be able to successfully establish and become widespread with very few individuals introduced to a new area, possibly even a single mated female. Though a single female of *X. sonorina* is now known from British Columbia, no subsequent work has confirmed its establishment in Canada (see Sheffield and Heron 2019). Considering the nesting and wintering biology described for this species by Giffard (1922), Williams (1927) and Gerling (1982) [material from Hawaii], it is possible that several individuals were transported from the continental United States to these Pacific Islands, or perhaps it island-hopped from an initial establishment event in Hawaii, where the species has occurred since at least the mid-1870s (Smith 1874). Interestingly, this species also seems to have made it as far west as Java in Indonesia (Cockerell 1919) and more recently, on at least two occasions, New Zealand (Donovan 1988, Manson 1988). Donovan (2007) indicated that only males were found in the nests found in New Zealand, and suggested this was likely due to the egg laying female not being mated pre-arrival, suggesting that perhaps an unmated female and not an established or wintering nests of contain both males and females arrived. Thus, the reproductive status of immigrant populations, and the ability to take advantage of new habitat likely influence successful establishment of bees around the globe.

## Supplementary Material

XML Treatment for Xylocopa (Neoxylocopa) sonorina

## Figures and Tables

**Figure 1a.**
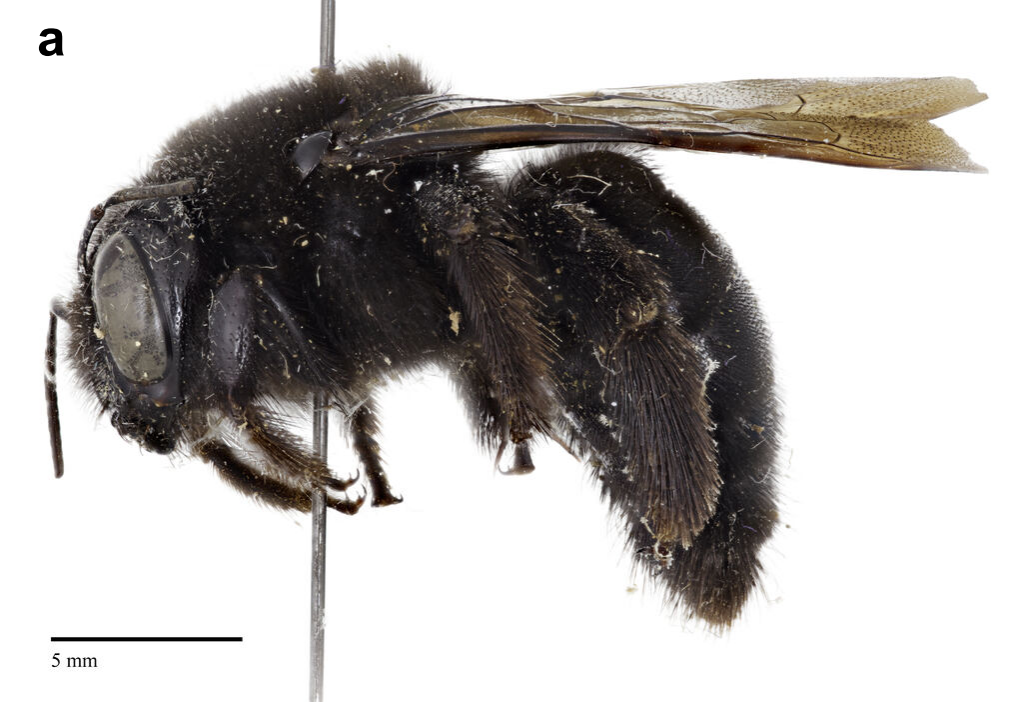
Lateral view

**Figure 1b.**
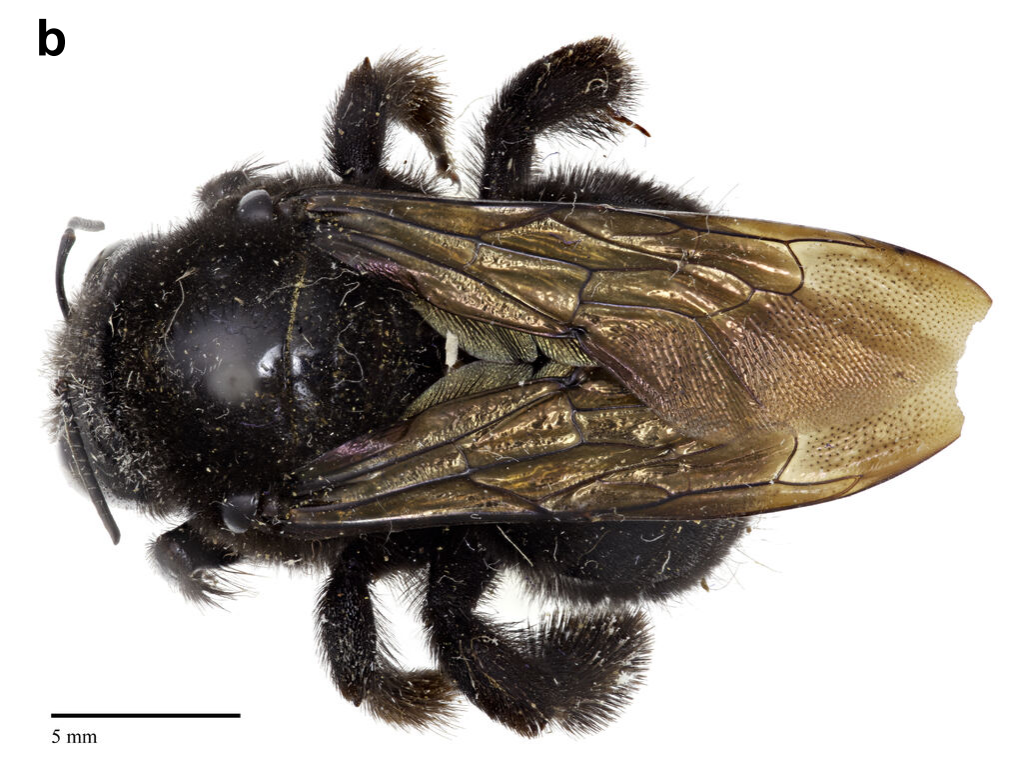
Dorsal view

**Figure 1c.**
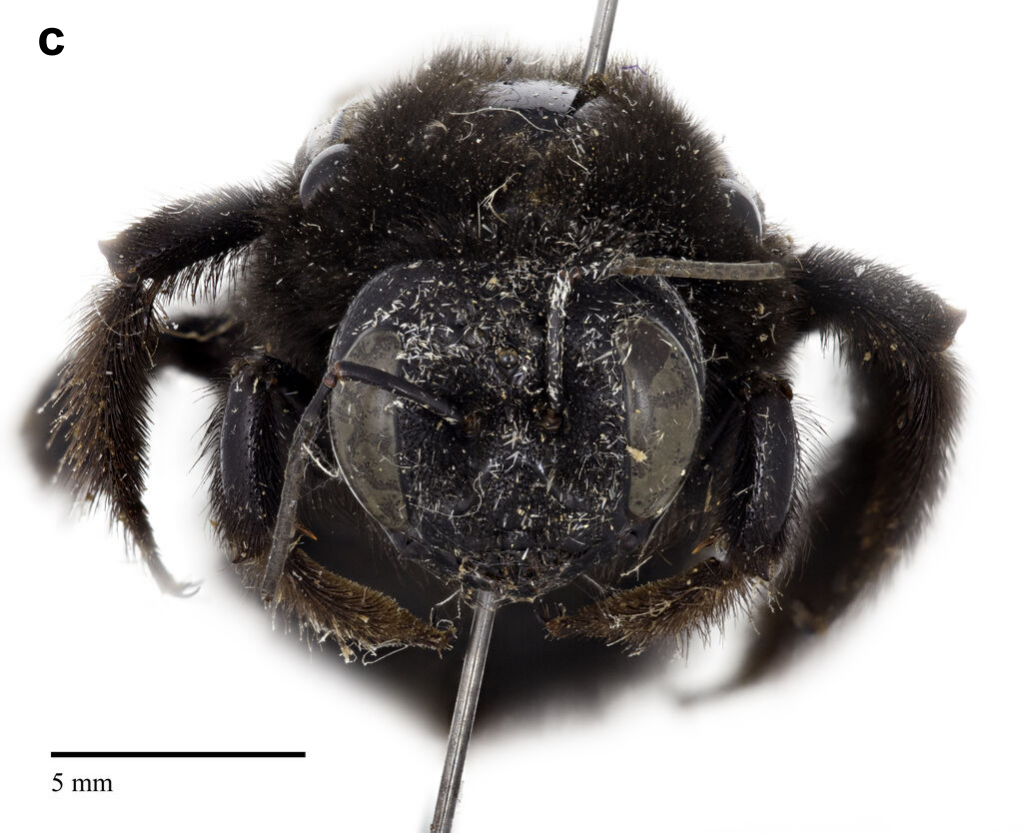
Frontal view

**Figure 1d.**
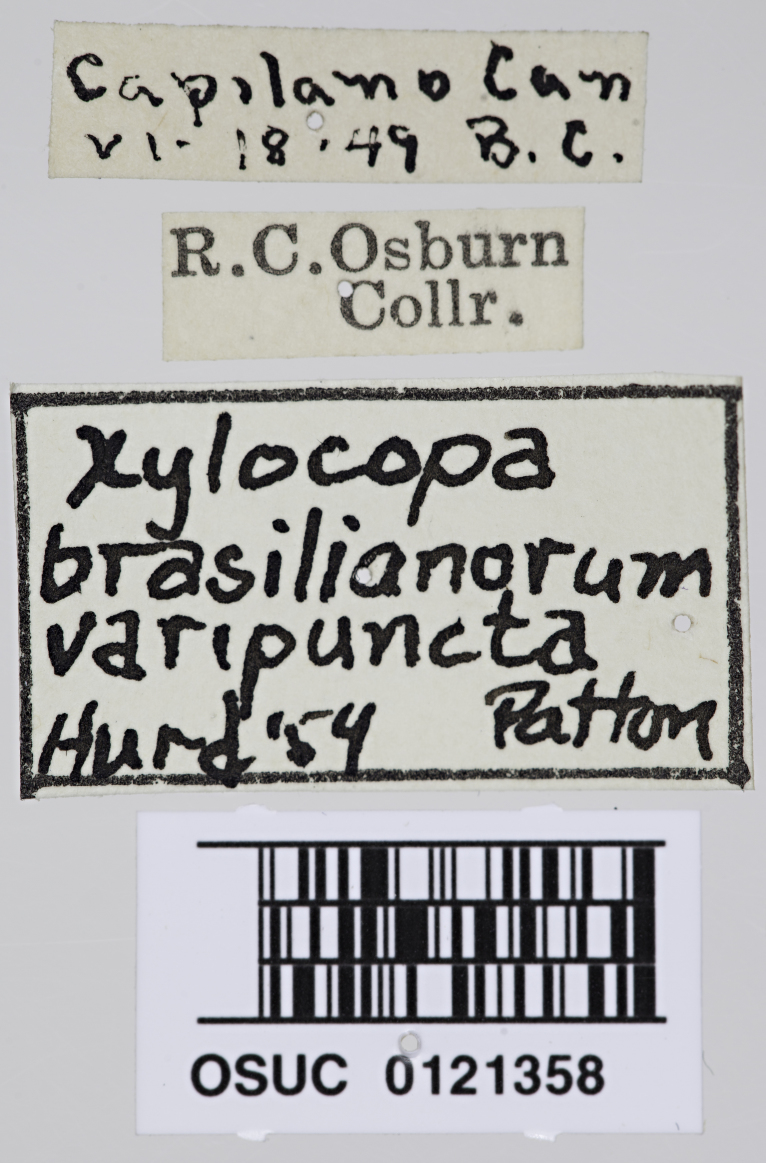
Specimen labels
